# Supplementary figures and images for: LANA-dependent transcription-replication conflicts and R-loops at the terminal repeats (TR) correlate with KSHV episome maintenance
Source: PLoS Pathog. 2025 Aug 18;21(8):e1013029. doi: 10.1371/journal.ppat.1013029 (PMC12396754; doi:10.1371/journal.ppat.1013029)

Supplementary Figure 1

A

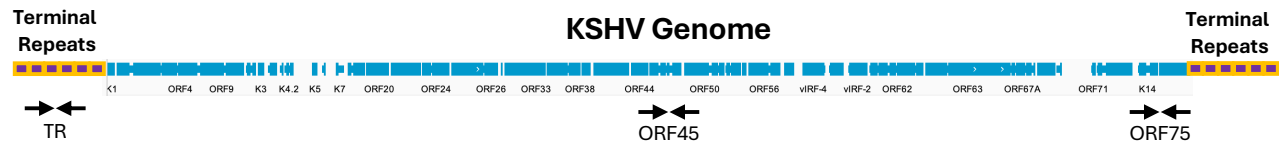

B

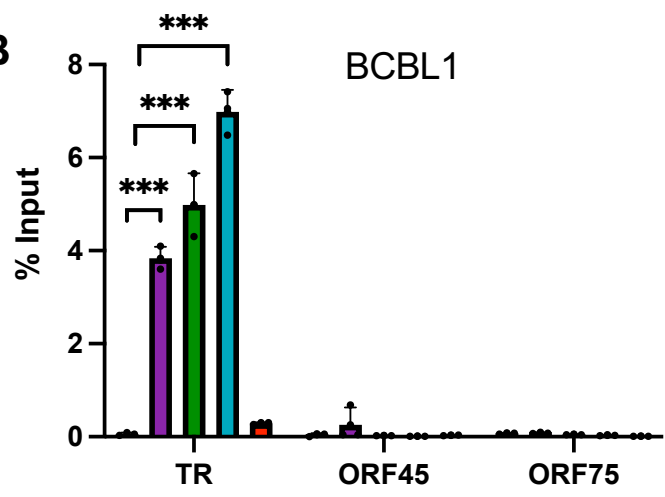

C

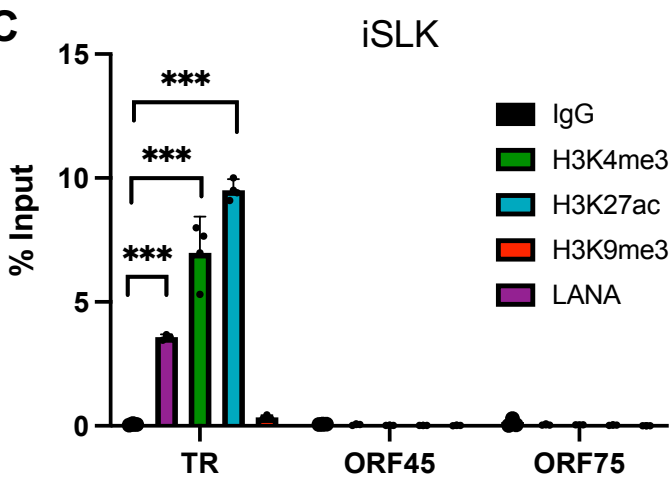

D

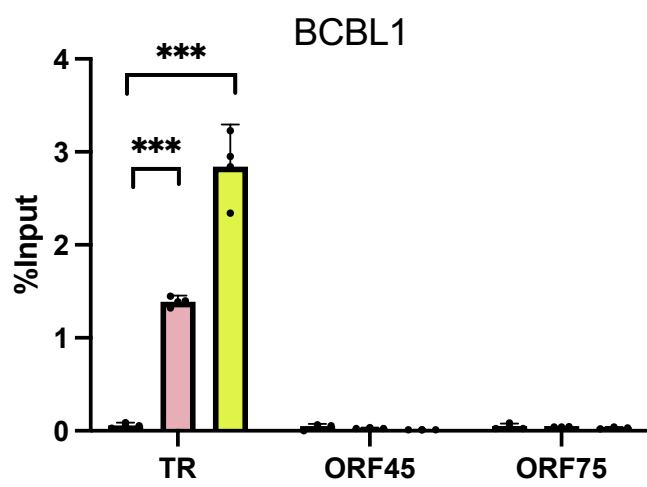

E

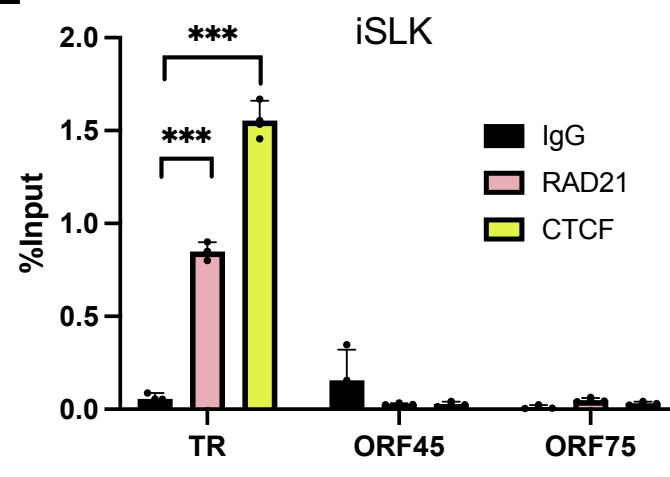

Supplement: S1 Fig — A. Schematic of the KSHV genome showing the terminal repeats (TR) relative to the unique region open reading frames (blue) and primer positions for TR, ORF45, and ORF75. B. ChIP-qPCR for histone H3K4me3, H3K27ac, H3K9me3, LANA or control IgG assayed at the TR, ORF45 or ORF75 loci in BCBL1 or iSLK cells. C. Same as in panel B, except ChIP antibodies with RAD21, CTCF, or IgG control. ** p < .01, *** p < .001, student 2-tailed t-test, n = 3 biological replicates. (PDF) [file ppat.1013029.s001.pdf]

Supplementary Figure 2

A

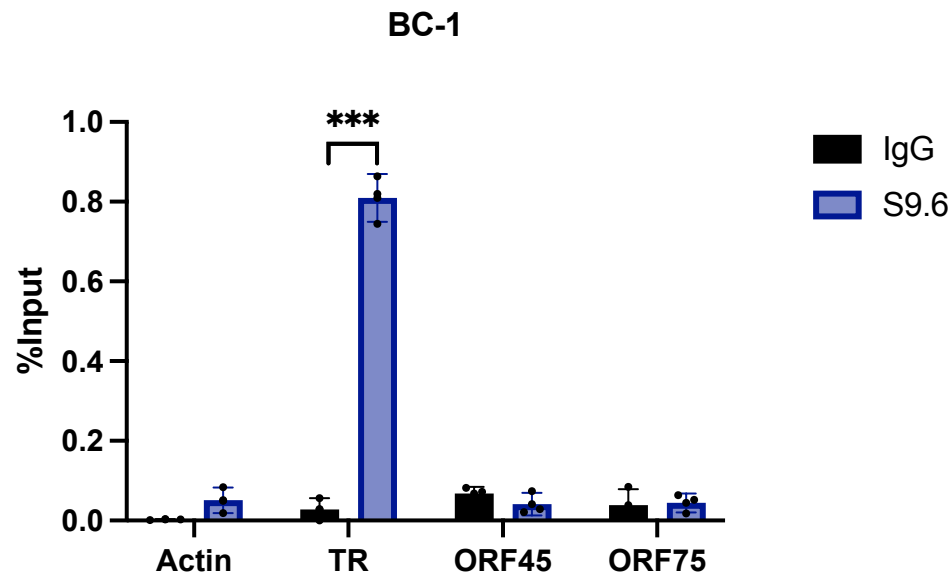

B

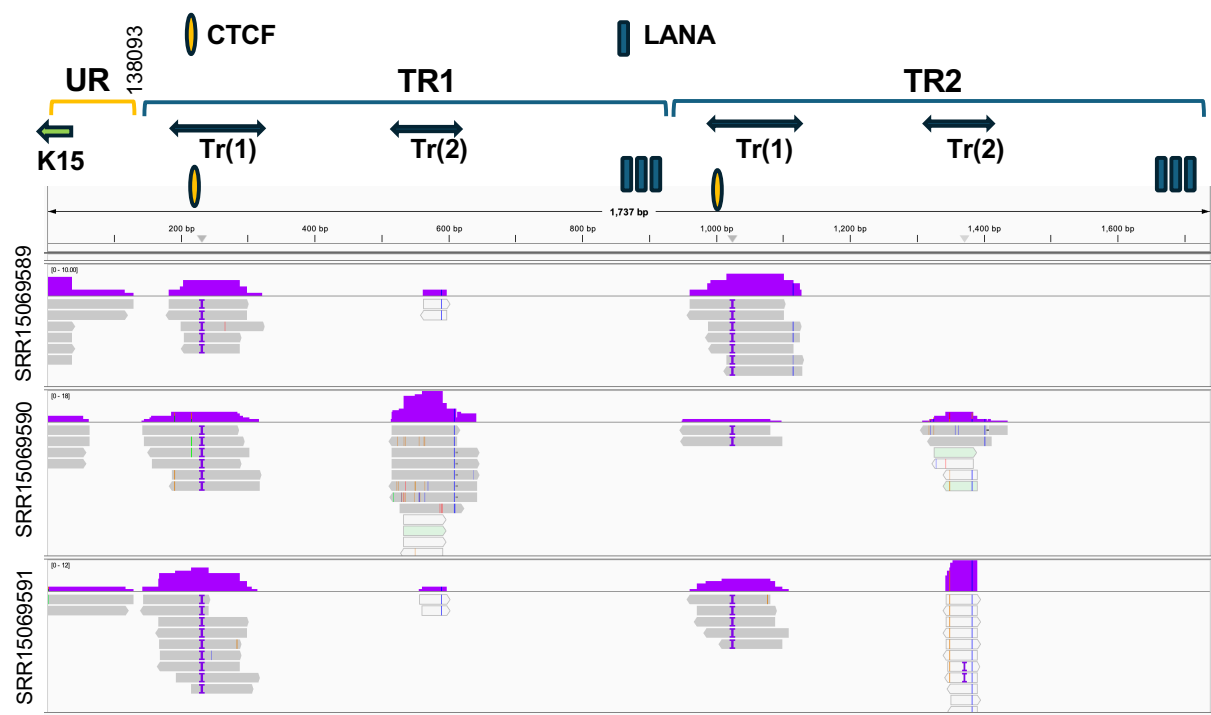

Supplement: S2 Fig — A. DRIP assay with BC1 cells using S9.6 (blue) or control IgG (black) assayed with primers for cellular actin or KSHV TR ORF45 and ORF75. *** p < .001, student two-tailed t-test. B. IGV screen shot of RNA transcripts mapped to KSHV TR region using public data sets for total RNAseq in BCBL1 cells during latent conditions (SRR15069589, SRR15069590, SRR15069591). The reference map consists of a small region of the unique region with K15 and 2 copies of the TR. TR transcripts Tr(1) and Tr(2) are indicated above. (S2_Fig.PDF) [file ppat.1013029.s002.pdf]

## Supplementary Figure 3

**A**

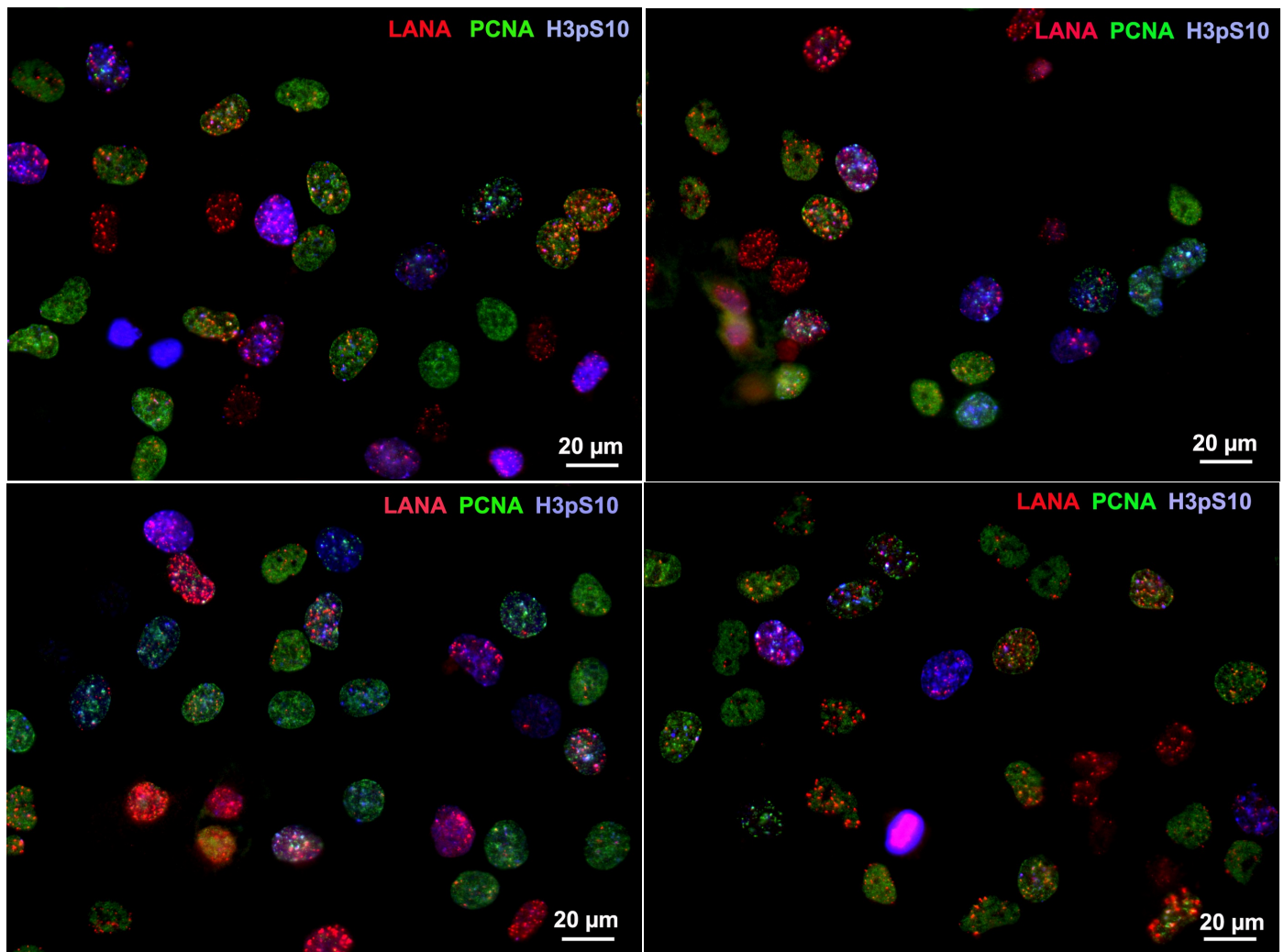

**B**

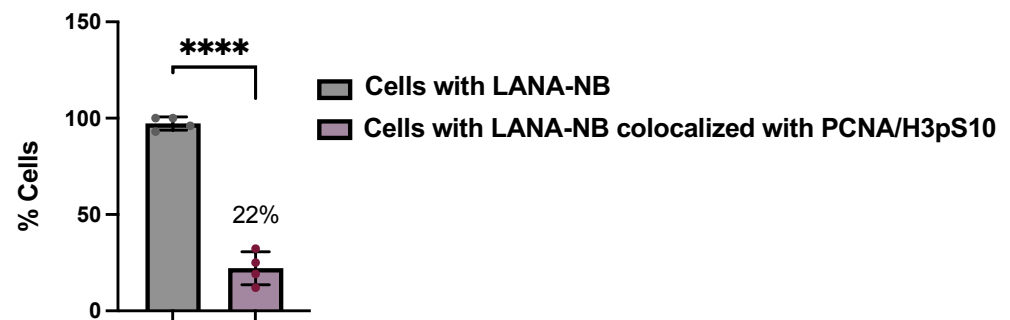

Supplement: S3 Fig — A. Representative images of iSLK cells showing the percentage of cells with LANA and colocalizations with H3pS10 and PCNA. 60x magnification, N = 4, total of 126 cells, B. Quantification of percentage of cells with LANA-NB or LANA-NBs colocalized with both PCNA and H3pS10. ****p < .0001, student two-tailed t-test. (S3_Fig.PDF) [file ppat.1013029.s003.pdf]

Supplementary Figure 4

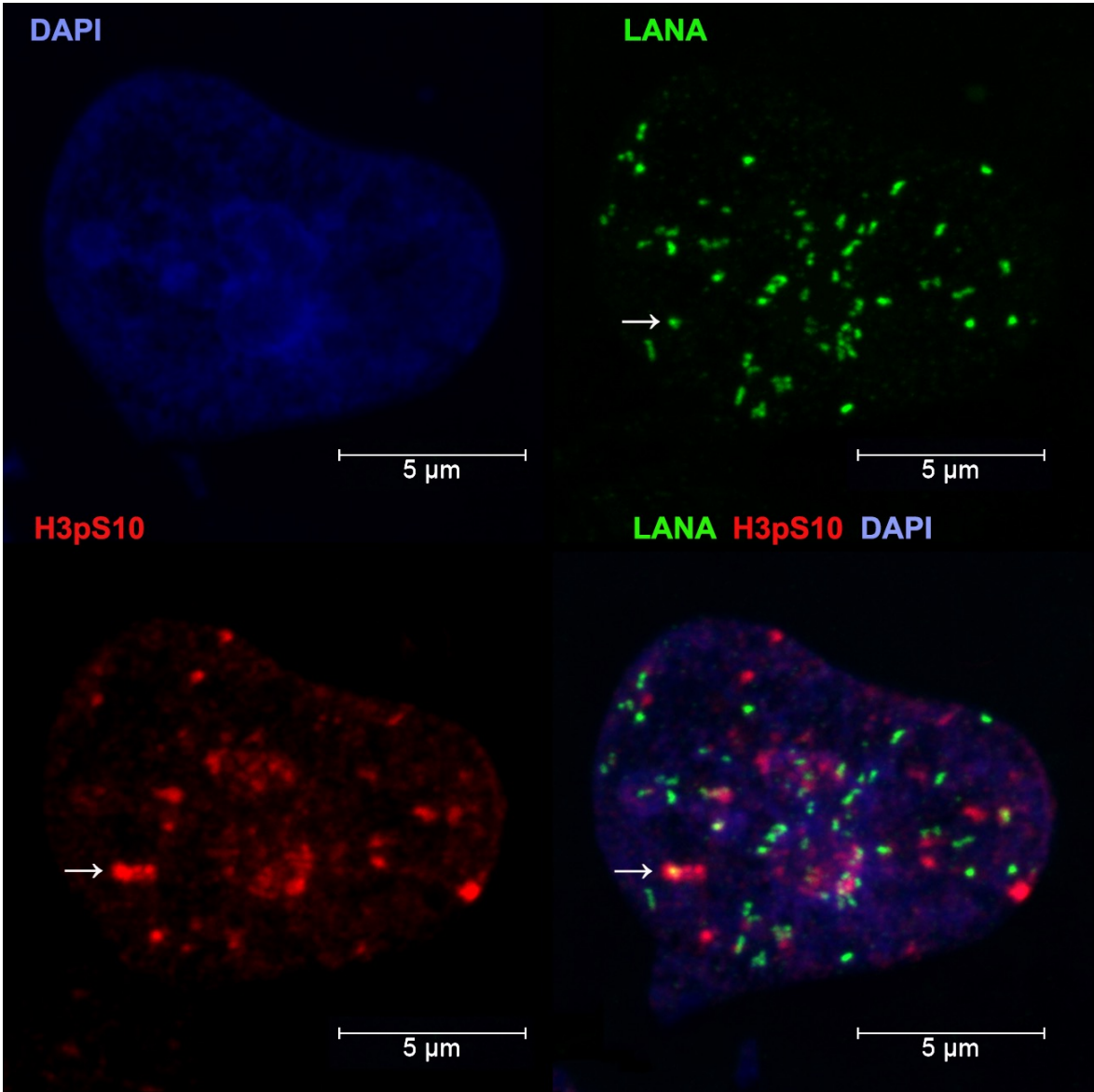

Supplement: S4 Fig — H3pS10 (blue), LANA (red), Dapi (blue). Arrows indicate examples of colocalization. (S4_Fig.PDF) [file ppat.1013029.s004.pdf]

Supplementary Figure 5

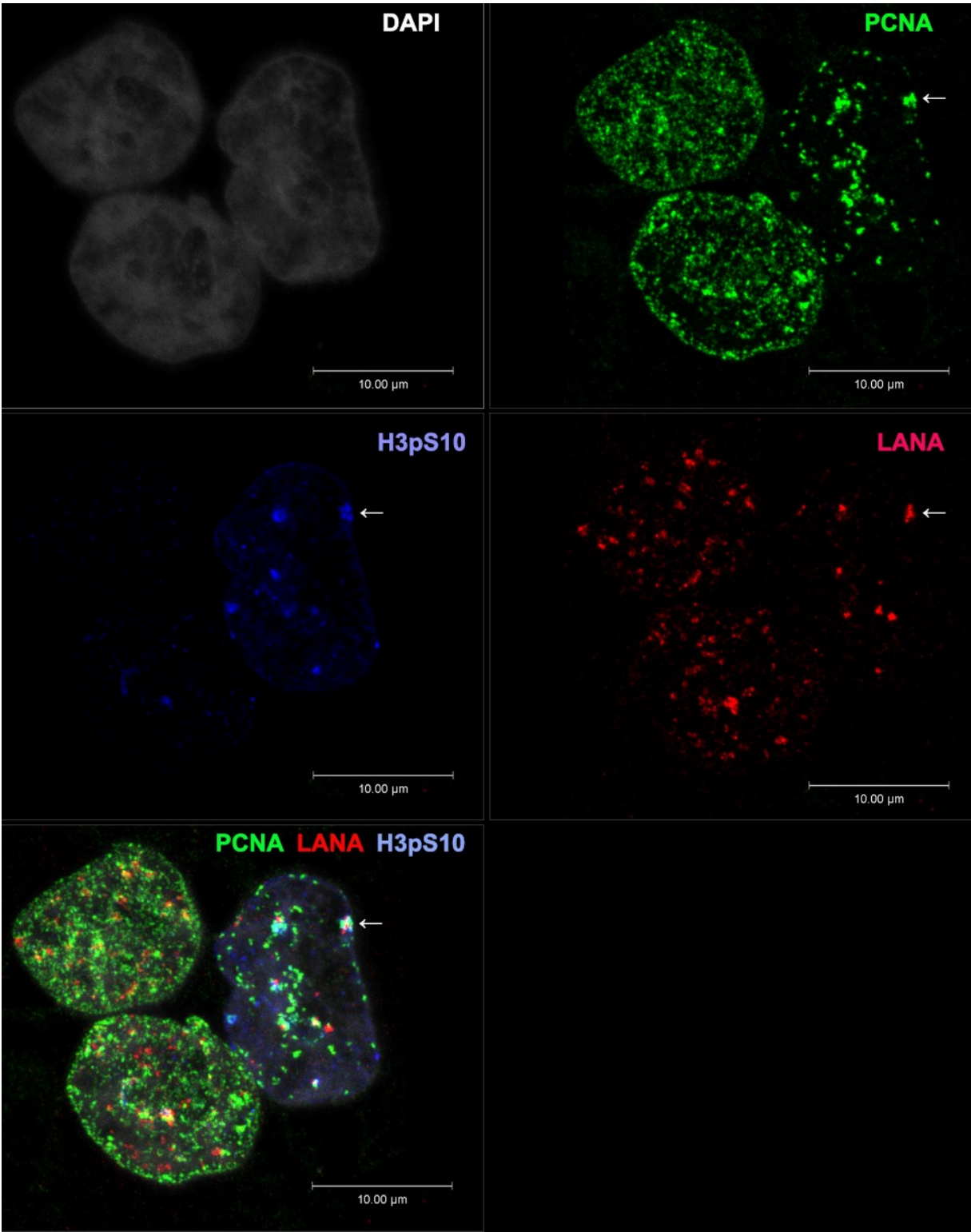

Supplement: S5 Fig — PCNA (green), H3pS10 (blue), LANA (red). Arrow indicates example of colocalization. (S5_Fig.PDF) [file ppat.1013029.s005.pdf]

Supplemental Figure 6

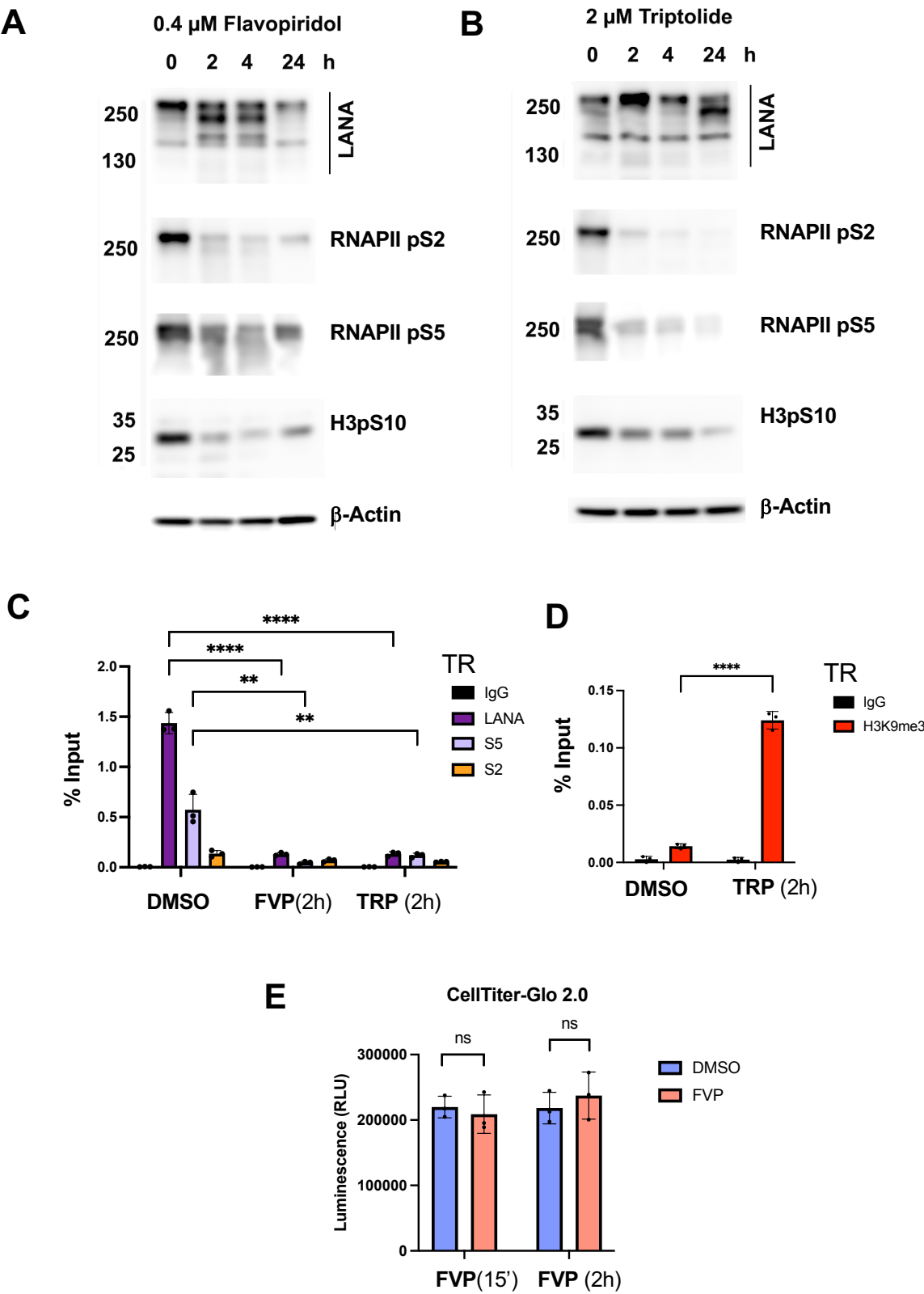

Supplement: S6 Fig — A-B. Western blots of BCBL1 cells treated with 0.4 μM FVP (pane A) or with 2 μM triptolide (panel B) for 0, 2, 4 or 24 hrs and probed for LANA, RNAPII pS2, pS5, H3pS10, or β-actin. C. ChIP-qPCR for IgG, LANA, RNAPII pS5 or pS2 in BCBL1 cells treated with either FVP or triptolide (TRP) for 2 h. D. Same as in panel C, except for ChIP-qPCR with IgG and H3K9me3. **p < .01, ****p < .0001, student two-tailed t-test. (S6_Fig.PDF) [file ppat.1013029.s006.pdf]

Supplemental Figure 7

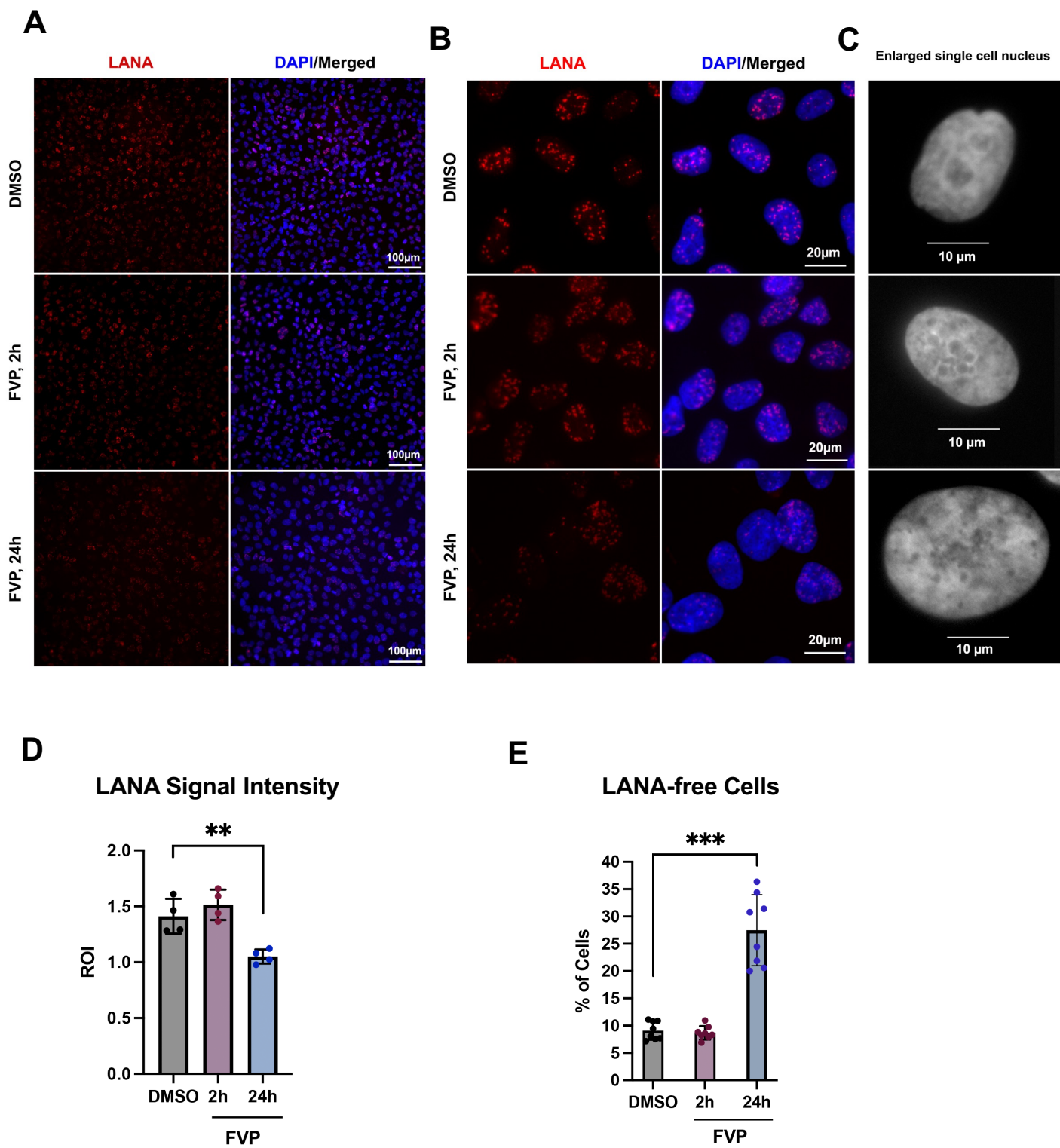

Supplement: S7 Fig — iSLK cells were treated with DMSO, 0.4 μM FVP for 2h or 24 hr and assayed by IF using LANA antibody (red) and counterstained with Dapi (blue) imaged at 20x (panel A), 60x (panel B), or enlarged single nuclei imaged by phase contrast (panel C). D. Quantification of LANA signal intensity. E. Quantification of cells lacking LANA signals (LANA-free cells). **p < .01, ***p < .001, student two-tailed t-test. (S7_Fig.PDF) [file ppat.1013029.s007.pdf]

Supplemental Figure 8

A

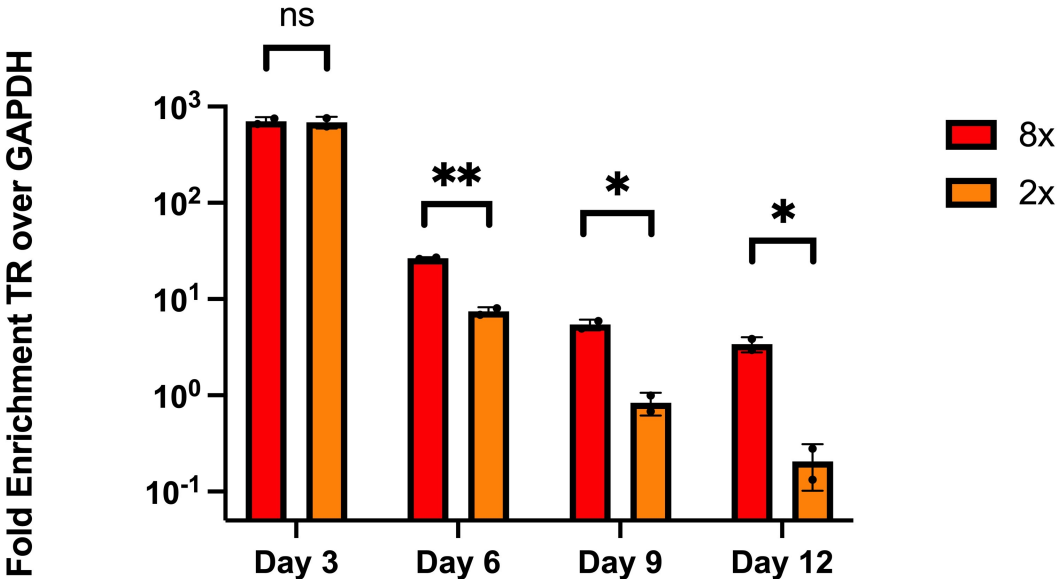

B

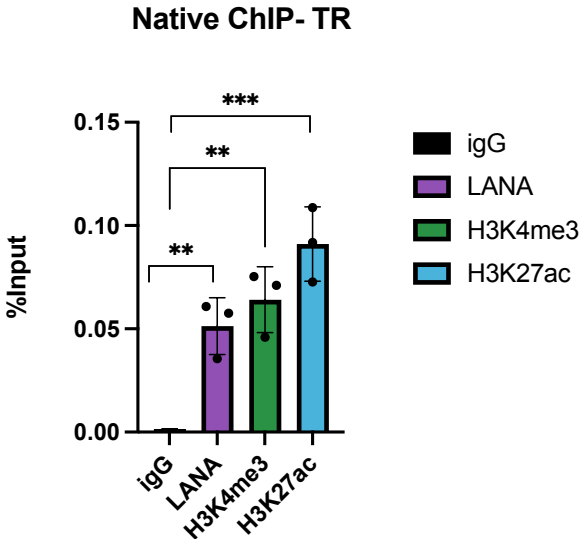

Supplement: S8 Fig — A. TR DNA was quantified by qPCR in 293T cells transfected with F-LANA and either p8xTR or p2xTR plasmids at days 3, 6, 9, and 12. *p < .05, **p < .01, student two-tailed t-test. B. Native ChIP for IgG, LANA, H3K4me3, H3K27ac in BCBL1 cells using conditions identical to standard ChIP assay but lacking initial formaldehyde cross-linking. (S8_Fig.PDF) [file ppat.1013029.s008.pdf]

Supplemental Figure 9

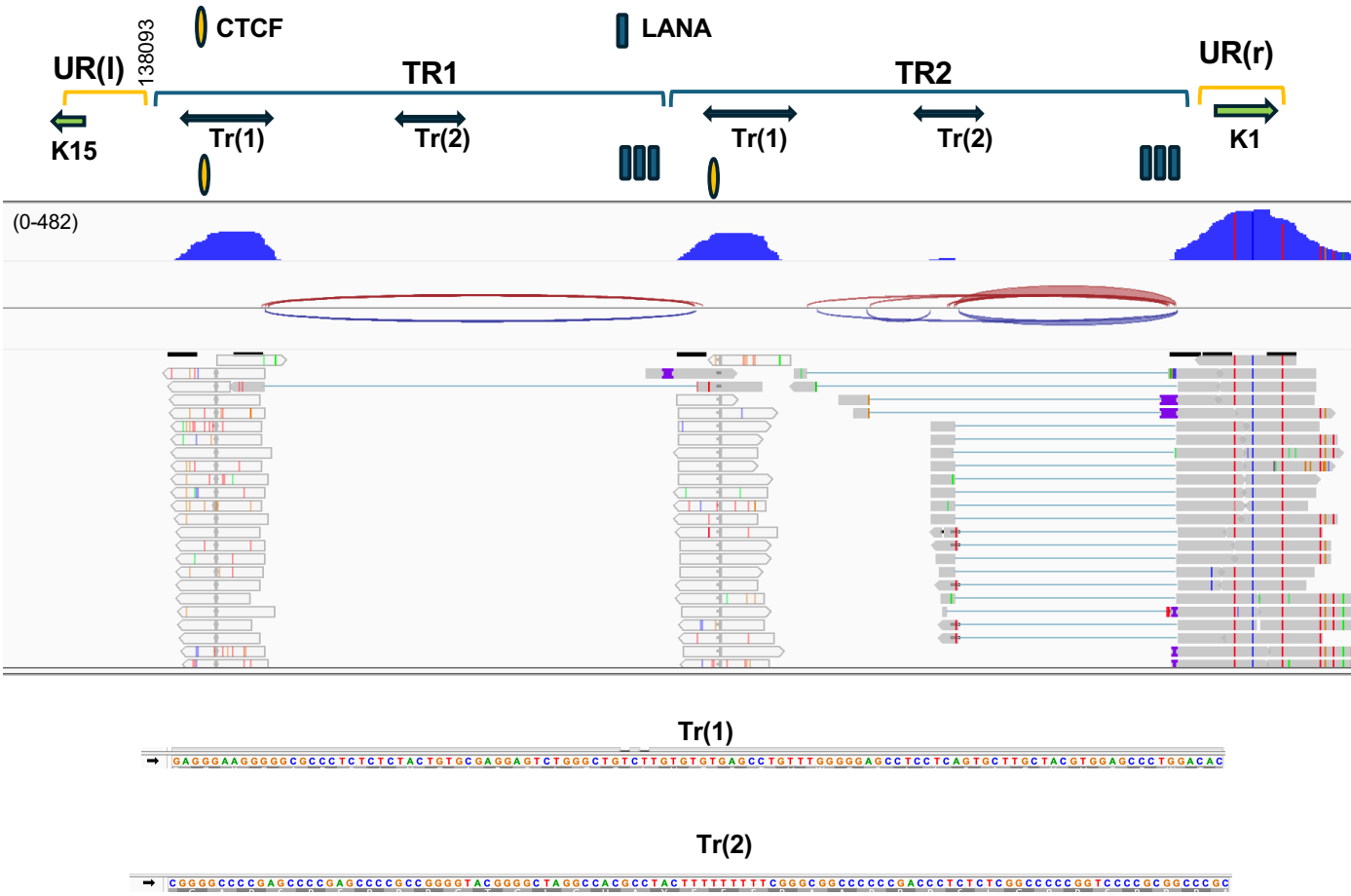

Supplement: S9 Fig — 29T cells transfected with p8xTR and pFLAG-LANA were assayed 72 hrs post-transfection for total RNAseq using Illumina paired-end method. FASTQ files were mapped to the KSHV subgenomic fragment containing K15-2xTR-K1 sequence and visualized using IGV genome browser. LANA and CTCF binding sites are indicated above. Blue peaks represent read counts and presumptive splice junctions are indicated by blue and red loops. Sequence of transcripts Tr (1) and T2 (2) are indicated below. (S9_Fig.PDF) [file ppat.1013029.s009.pdf]

Supplemental Figure 10

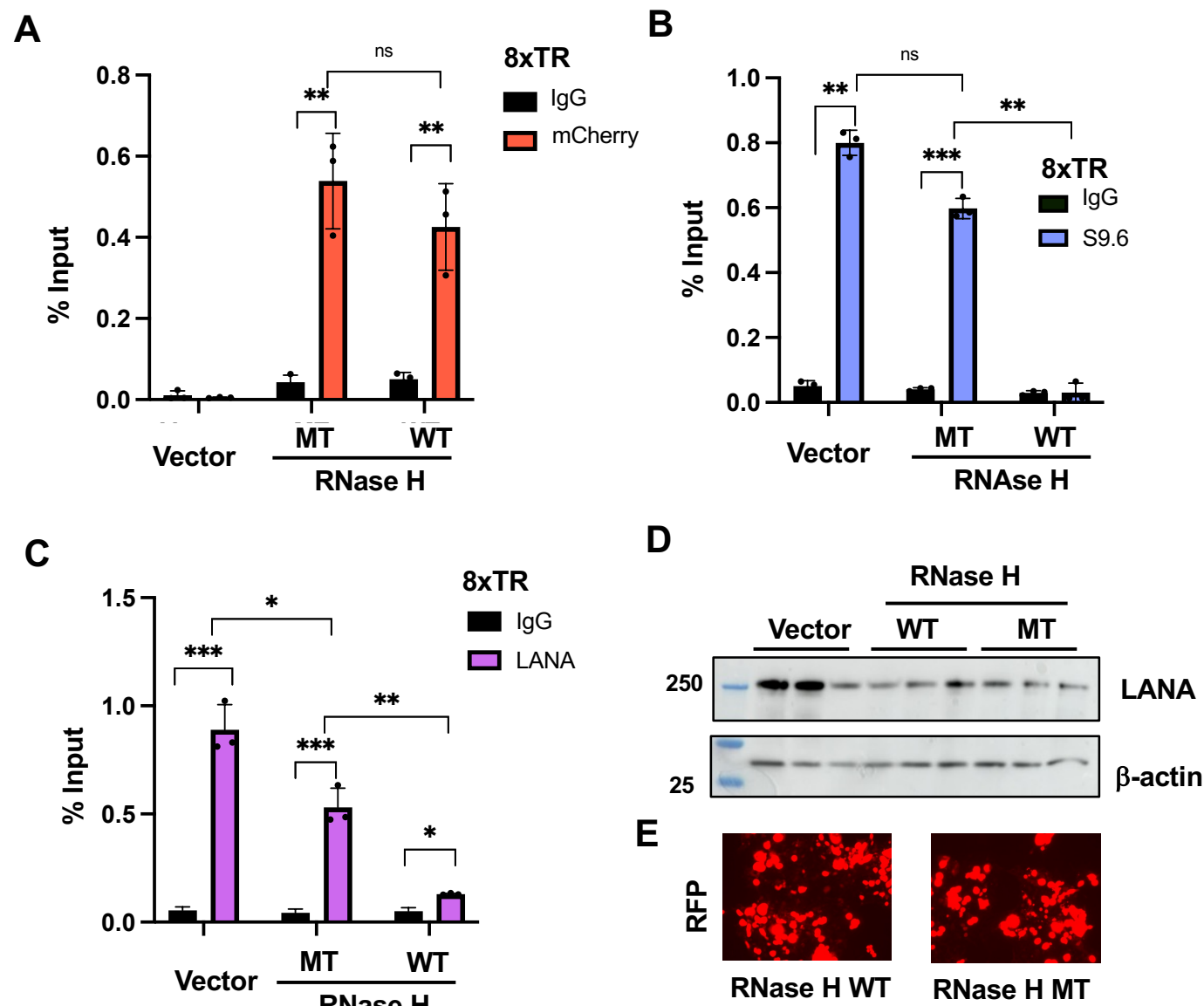

Supplement: S10 Fig — A-C. mCherry-tagged RNase H WT or MT or empty vector were co-transfected with p8xTR + FLAG-LANA and assayed for binding to the TR region by ChIP assay using antibody mCherry (panel A) and TR- DRIP assay (panel B), or LANA ChIP assay (panel C) and assayed with primers specific for KSHV TR. *p < .05, **p < .01, ***p < .001, or not significant (ns) using student two-tailed t-test. D. Western blot of 3 biological replicates used for experiments shown in panels A-C. E. Fluorescence microscopy of RFP for RNAse H WT and RNAse H MT expression in transfected 292T cells. (S10_Fig.PDF) [file ppat.1013029.s010.pdf]
