# Supplementary material for: LANA-dependent transcription-replication conflicts and R-loops at the terminal repeats (TR) correlate with KSHV episome maintenance
Source: PLoS Pathog. 2025 Aug 18;21(8):e1013029. doi: 10.1371/journal.ppat.1013029 (PMC12396754; doi:10.1371/journal.ppat.1013029)
Supplement: S1 Table — (S1_Table.PDF) [file ppat.1013029.s012.pdf]

Supplemental Table 1. Oligonucleotide Primers

ChIP-qPCR

CCTGCCGGGGACGCCGCCGGGGCCT	LBS_5'
CTGAGGCGGCGCGCGGCCCCAT	LBS_3'
TATACAAGCCGTACGGGCAC	ORF75F
CTCGAAGTGGGAGGTCTTCG	ORF75R
GCTTTGCGGCTTAAGTTTGG	ORF45R
CGCCTCCTCTGGTAGCGA	ORF45F
ACATTTGCCCCACCGTCGCCT	ORF16F
GCACATAGCACGCGCACAGCA	ORF16R

RT-qPCR

GACCCCGGGCAGCGAGGGAA	TR pr1 f
AGGGCTCCACGTAGCAAGCACTG	TR pr1 r
CCTGCCGGGGACGCCGCCGGGGCCT	TR pr2 f
CTGAGGCGGCGCGCGGCCCCAT	TR pr2 r
