# Supplementary material for: LANA-dependent transcription-replication conflicts and R-loops at the terminal repeats (TR) correlate with KSHV episome maintenance
Source: PLoS Pathog. 2025 Aug 18;21(8):e1013029. doi: 10.1371/journal.ppat.1013029 (PMC12396754; doi:10.1371/journal.ppat.1013029)
Supplement: S1 File — (DOCX) [file ppat.1013029.s014.docx]

**Supporting Information_ Legend for PRISM files**

Fig.1B- ChIP-qPCR for RNAP II pS5, RNAPII pS2, MCM2, PCNA or control IgG assayed at the TR, ORF45 or ORF75 loci in BCBL1 cell line.

Fig.1C-ChIP-qPCR for RNAP II pS5, RNAPII pS2, MCM2, PCNA or control IgG assayed at the TR, ORF45 or ORF75 loci iSLK cell line.

Fig1.E-Quantification of IF images showing % colocalization of PLA signal with LANA-NBs.

Fig.2A- DRIP assay with BCBL1 cells using S9.6 (blue) or control IgG (black) assayed with primers for cellular actin, KSHV TR, ORF45 or ORF75.

Fig. 2B DRIP assay treated with RNase H controls.

Fig. 3A- ChIP-qPCR for LANA, H3pS10 and IgG at the TR, ORF45, ORF50, and ORF75 loci of KSHV in BCBL1 cells.

Figure 3B- ChIP-qPCR for RNAPII pS2, MCM, H3pS10 during different stages of the cell cycle using centrifugal elutriation for G1, G1/S, S, Late S, G2, and G2/M, TR primers.

Figure 3C- ChIP-qPCR for RNAPII pS2, MCM, H3pS10 during different stages of the cell cycle using centrifugal elutriation for G1, G1/S, S, Late S, G2, and G2/M Orf16 primers.

Fig.4B- Quantification of the percent of LANA-NBs colocalized with PCNA.

Fig. 4D; Quantification of the percentage of LANA-NBs colocalized with H3pS10.

Fig. 4F- Quantification of the percentage of PCNA foci colocalized with H3pS10.

Fig. 4H- Quantification of the percentage of LANA-NBs colocalized with H3pS10 and PCNA.

Fig. 5B- ChIP-qPCR for IgG, LANA, RNAPII pS2 or pS5 at the TR in BCBL1 cells treated with DMSO or FVP for 15’ or 2h.

Fig. 5C- DRIP assay with S9.6 or IgG for BCLB1 cells treated with DMSO or FVP for 15’ or 2h;

Fig.5D- **.** ChIP-qPCR for H3K4me3, H3K9me3, H3K27ac or IgG in BCBL1 cells treated with DMSO or FVP for 15’ or 2h.

Fig.5E- ChIP-qPCR for LANA or total histone H3 in BCBL1 cells treated with DMSO or FVP for 15’ or 2h; Fig.5F- ChIP-qPCR for LANA or IgG at TR in BCLB1 cells treated with DMSO, FVP or Triptolide for 15’.

Fig. 5G- ChIP-qPCR for CTCF, RAD21 or IgG at TR in BCLB1 cells treated with DMSO, FVP, or Triptolide for 15’; Fig.6C- ChIP-qPCR of 8xTR episome template with F-Vector or F-LANA assayed for LANA, H3K4me3, H3K9me3, H3K29ac or IgG.

Fig. 6D- ChIP-qPCR for 8xTR episome template with F-Vector of F-LANA assayed for LANA, RNAP II pS2, pS5, or IgG; Fig.6E- ChIP-qPCR with 2xTR or 8xTR template in the presence of F-LANA assayed for LANA, H3K4me3, H3K27ac, or IgG.

Fig.6F- ChIP-qPCR for 2xTR or 8xTR in presence of F-LANA assayed for RNAP II pS2, pS5 or IgG; Fig.6G; DRIP with S9.6 or IgG assayed at the TR for BCBL1, 8xTR with F-Vector, 2xTR with F-LANA or 8xTR with F-LANA.

Fig. 6H- RT-qPCR analysis of TR RNA using primers TR pr1 or TR pr2 from cells transfected with 2xTR or 8xTR and either F-Vector or F-LANA and normalized to GAPDH.

Fig.7C- ChIP-qPCR for IgG, LANA, H3K4me3, H3K9me3, or H3K27ac in 293T cells transfected with LANA-WT or LANA-LRSm assayed at the TR**.**

Fig. 7D- ChIP-qPCR for IgG, LANA, RNAP II pS2 and pS5 in 293T cells transfected with LANA-WT or LANA.

Fig.7E- DRIP assay in 293T cells transfected with LANA-WT or LANA-LRSm using IgG or S9.6 antibody assayed at the TR.

S.1B- ChIP-qPCR for histone H3K4me3, H3K27ac, H3K9me3, LANA or control IgG assayed at the TR, ORF45 or ORF75 loci in BCBL1 or iSLK cells.

S1.C- ChIP antibodies with RAD21, CTCF, or IgG control IN iSLK or BCBL1 cells. SS2.A- DRIP assay with BC1 cells using S9.6.

S3.B- , B. Quantification of percentage of cells with LANA-NB or LANA-NBs colocalized with both PCNA and H3pS10.

S6.C - ChIP-qPCR for IgG, LANA, RNAPII pS5 or pS2 in BCBL1 cells treated with either FVP or triptolide (TRP) for 2 h.

S6.D- ChIP-qPCR with IgG and H3K9me3 in BCBL1 cells treated with either FVP or triptolide (TRP) for 2 h; S7.D- Quantification of LANA signal intensity.

S7.E- Quantification of LANA-free cells; S8- TR DNA was quantified by qPCR in 293T cells transfected with F-LANA and either p8xTR or p2xTR plasmids at days 3, 6, 9, and 12.
